# West Nile Virus Seroprevalence Among Outdoor Workers in Southern Italy: Unveiling Occupational Risks and Public Health Implications

**DOI:** 10.3390/v17030310

**Published:** 2025-02-24

**Authors:** Angela Stufano, Valentina Schino, Gabriele Sacino, Riccardo Ravallese, Roberto Ravallese, Leonarda De Benedictis, Anna Morea, Roberta Iatta, Simone Giannecchini, Maria A. Stincarelli, Maria Chironna, Claudia Maria Trombetta, Piero Lovreglio

**Affiliations:** 1 Interdisciplinary Department of Medicine, Section of Occupational Medicine, University of Bari, 70124 Bari, Italy; angela.stufano@uniba.it (A.S.); sacinogabriele@gmail.com (G.S.); riccardo.ravallese@uniba.it (R.R.); roberto.ravallese@gmail.com (R.R.); leonarda.debenedictis@uniba.it (L.D.B.); anna.morea@uniba.it (A.M.); roberta.iatta@uniba.it (R.I.); piero.lovreglio@uniba.it (P.L.); 2 Department of Experimental and Clinical Medicine, University of Florence, 50134 Firenze, Italy; simone.giannecchini@unifi.it (S.G.); mariaalfreda.stincarelli@unifi.it (M.A.S.); 3 Interdisciplinary Department of Medicine, Section of Hygiene, University of Bari, 70124 Bari, Italy; maria.chironna@uniba.it; 4 Department of Molecular and Developmental Medicine, University of Siena, 53100 Siena, Italy; trombetta@unisi.it

**Keywords:** West Nile virus, outdoor workers, livestock, occupational medicine

## Abstract

Background: West Nile virus (WNV) is a mosquito-borne RNA virus, with birds as reservoirs and humans as incidental hosts. WNV often causes asymptomatic infections, but severe neuroinvasive disease occurs in fewer than 1% of human cases. Recent climatic changes and occupational exposure have increased its spread, particularly in Southern Italy. This study aimed to assess WNV seroprevalence and occupational risks among outdoor workers to guide targeted public health interventions. Methods: This cross-sectional study was conducted in the Apulia region, southeastern Italy, from November 2023 to April 2024. Participants completed a detailed questionnaire on socio-demographics, occupational exposure, travel history, and health symptoms. Blood samples were analyzed using enzyme-linked immunosorbent assay (ELISA) and neutralization assays to detect WNV-specific antibodies. Results: 250 outdoor workers in southeastern Italy were recruited, including agricultural workers, veterinarians, forestry workers, and livestock breeders. The latter showed the highest WNV prevalence at 6.5%. Protective measures such as repellent use (β = −0.145, OR = 0.95, *p* = 0.019) and personal protective equipment (PPE) usage (β = −0.12, OR = 0.94, *p* = 0.04) significantly reduced the likelihood of WNV infection. Conclusions: The study highlights the significant occupational risk posed by WNV to outdoor workers involved in livestock breeding in Southern Italy, likely due to their frequent exposure to mosquito-prone environments. Tailored public health strategies and education programs are needed to protect high-risk outdoor workers from WNV, amidst the backdrop of changing climatic conditions that favor increased transmission.

## 1. Introduction

West Nile virus (WNV) is an emerging single-stranded RNA virus belonging to the genus *Orthoflavivirus* (Family *Flaviviridae*). It is classified within the Japanese encephalitis serocomplex [1]. WNV exists in two main genetic lineages: lineage 1 (WNV-1), which is prevalent across the Americas, North Africa, Europe, and Australia, and lineage 2 (WNV-2), which is endemic to South Africa and Madagascar and has also been present in Europe since 2004 [2].

The primary mode of transmission for WNV is through the bite of infected *Culex* mosquitoes, with birds acting as natural reservoirs that amplify the virus in the environment. Mammals, including humans and equids, are incidental hosts of WNV [3]. While they can develop clinical signs upon infection, the severity and frequency of symptoms vary. In humans, the majority of infections remain asymptomatic. However, in approximately 20–30% of cases, particularly among the elderly or immunocompromised individuals, flu-like symptoms collectively referred to as West Nile fever (WNF) [4] may develop after an incubation period of 3–14 days. Mosquito bites are the primary route of infection, though rare cases of human-to-human transmission have been reported through organ transplantation, blood transfusions, breastfeeding, transplacental transmission, and occupational exposure in laboratory settings [4,5]. Severe cases can develop into complications such as hepatomegaly, splenomegaly, myocarditis, pancreatitis, or hepatitis. Fewer than 1% of infections progress to West Nile neuroinvasive disease (WNND), which can manifest as meningitis, encephalitis, or flaccid paralysis and may result in fatal outcomes [3].

Both biotic factors like bird migration and mosquito activity, and abiotic factors such as local climate conditions, significantly influence the transmission dynamics of WNV [6]. Climate change can affect the biological cycles of vectors and the abundance of animal reservoirs, potentially increasing WNV infection incidence [7,8]. In recent decades, Europe, including Italy, has experienced an increased frequency, geographic spread, and incidence of WNV outbreaks in humans and equids [9,10].

Between 2012 and 2020, Italy reported 1145 WNV infections, including 487 cases with neurological symptoms across 11 regions [11]. Since the start of the 2023 transmission season, Italy has recorded an increasing number of human WNV infections, with 439 cases identified [12]. While northern Italian regions were already considered endemic for WNV, the Apulia region saw a sharp increase in WNV infections between July and October 2023, marking a 10-year high since the first autochthonous case. Consequently, the Apulia region’s risk classification changed from low to high. During surveillance from January to October 2023, eight cases of WNV infection were identified in the Apulia region, with an estimated notification rate of 0.2 per 100,000 cases. All were reported between July and October, with six cases presenting as WNND [13]. By early May 2024, 452 confirmed cases of WNV infection had been reported in Italy, including 271 neuro-invasive cases (4 in Apulia) and 46 asymptomatic cases in blood donors [14].

A growing concern in WNV epidemiology is the risk of occupational exposure to mosquito bites, particularly among outdoor workers [5,15]. However, comparative seroprevalence studies involving different outdoor occupational groups are limited, making it challenging to quantify the additional risk posed by occupational exposure. Moreover, outdoor workers have not been systematically investigated, despite potentially facing a heightened risk of exposure to mosquito bites. Given these concerns, this study aimed to assess the seroprevalence of WNV and associated occupational risk factors among outdoor workers in Southern Italy.

## 2. Materials and Methods

### 2.1. Study Design

**Figure 1:**
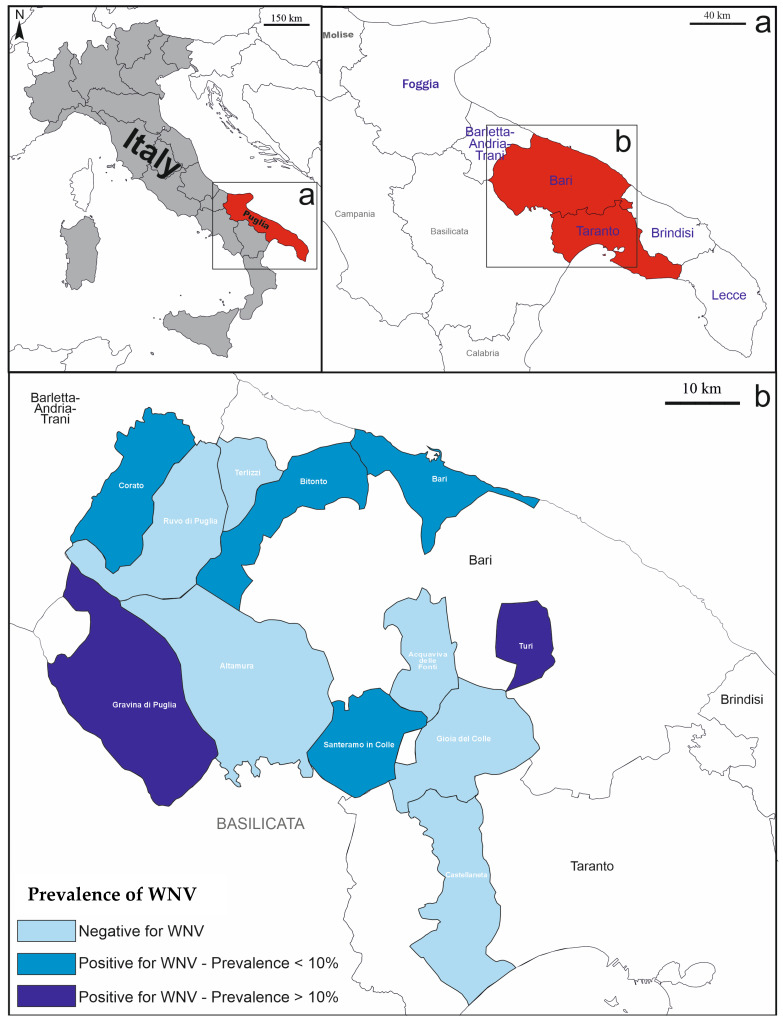
(**a**) Major municipalities where the seroprevalence study was carried out. (**b**) WNV seroprevalence rates among the recruited workers, for each single municipality of the investigated area.

This cross-sectional study was conducted between November 2023 and April 2024 in Apulia, located in southeastern Italy, and characterized by a Mediterranean climate with hot, dry summers and mild, wet winters [16]. In Figure 1, the specific municipalities where the workers were recruited are shown.

A minimum sample size of 85 subjects was determined a priori, based on the WNV prevalence reported in a previous study involving a population recruited in the same area [13]. Calculations assumed a 2% margin of error and a 95% confidence interval to ensure robust sampling and statistical validity for this population.

### 2.2. Occupation Selection and Workers’ Recruitment

The occupations selected for this study, forestry workers, livestock handlers, agricultural workers, veterinarians, and horse breeders, were chosen due to the workers’ potential high levels of outdoor activity and frequent encounters with mosquito habitats, both of which may potentially increase WNV exposure risk.

Recruitment of forestry workers, farmers, and livestock handlers occurred during educational meetings held across the Apulian region, in the course of which the study objectives and the WNV risks associated with mosquito bites were discussed. Enrolled forestry workers spent significant time in wooded and rural areas, maintaining forest health and clearing undergrowth. Farmers were involved in crop production, including water management practices, such as maintaining irrigation channels or collecting rainwater. Livestock handlers had contact with different types of animals. They performed a variety of daily activities centered on the care and management of livestock but also involving environmental maintenance, such as cleaning shelters, managing waste, and repairing fences or growing shelters.

Veterinarians primarily working with livestock and large animals in outdoor or partially open farm settings were invited to participate through voluntary health promotion programs aligned with Italian regulations for worker health protection (D. Lgs. 81/2008).

Additionally, a targeted group of military horse breeders was recruited at a specialized equestrian selection centre where a confirmed equine WNV infection had been detected about 6 weeks before the beginning of the study.

Participants had to meet the following inclusion criteria: to be at least 18 years old, to have been working in their respective occupation for at least 1 year, and to typically spend at least one-third of their daily working time in outdoor environments. Additionally, they were required to have no known history of immunodeficiency or prior confirmed WNV infection.

### 2.3. Questionnaire

Each participant completed a structured questionnaire to collect information on

1. socio-demographic background: age, sex, and education.
2. occupational exposure: job characteristics, working seniority, specific tasks performed, and the proportion of time spent working outdoors, history of exposure to mosquitoes at work and during leisure activities, use of repellents and personal protective equipment (PPE).
3. travel and vaccination history: recent travel to WNV-endemic areas, as well as vaccinations for other arboviruses like tick-borne encephalitis virus (TBEV), yellow fever virus (YFV), and Japanese encephalitis virus (JEV).
4. health history and symptoms: any previous infections with flaviviruses, and any symptoms compatible with WNF or WNND, experienced between April and November 2023.
5. Geographical location of working areas and worker’s residences.
6. Other outdoor activities such as camping, gardening, hunting.

The study was conducted following the Declaration of Helsinki and was consistent with ethical public health practice; it was approved by the ethics committee of the University Hospital of Bari (Italy) (approval no. 7770, protocol no. 0053676-08062023).

### 2.4. Serological Examination

For each participant, a 10 mL blood sample was collected in a Vacutainer tube. Serum samples were obtained after centrifugation at 3000× *g* for 10 min and stored at −20 °C until analysis.

All samples were tested by commercial ELISA (CHORUS West Nile Virus IgG; DIESSE- Diagnostica Senese S.p.A., Monteriggioni (SI), Italy) to detect IgG antibodies against WNV. Testing was performed according to the manufacturer’s instructions. Sera that scored IgG positive or borderline were further tested for the detection of IgM antibodies against WNV (CHORUS West Nile Virus IgM; DIESSE, Monteriggioni, Italy) and IgG antibodies against Dengue, Zika, Toscana and Chikungunya viruses (CHORUS Dengue VIRUS IgG; DIESSE, Monteriggioni, Italy; CHORUS Zika VIRUS IgG; DIESSE, Monteriggioni, Italy; CHORUS Toscana Virus IgG; DIESSE, Monteriggioni, Italy; CHORUS Chikungunya Virus IgG; DIESSE, Monteriggioni, Italy) to evaluate possible cross-reactivity between other arboviruses.

For microneutralization (MN) and plaque reduction neutralization (PRN) tests, Vero E6 (African green monkey kidney cell line; ATCC^®^ CRL-1586™) propagated in Dulbecco’s Modified Eagles Medium (DMEM; Sigma-Aldrich, St. Louis, MO, USA) supplemented with 10% Fetal Bovine Serum (FBS; Sigma-Aldrich, St. Louis, MO, USA) were used. The WNV strain (lineage 2) viral stock, derived from cell-free supernatants of acutely infected Vero E6 cells, was titrated for a 50% tissue culture infectious dose (TCID_50_) and plaque-forming unit (PFU) in Vero E6 cells and stored at −80 °C until used. All serum samples were heat-inactivated at 56 °C for 30 min. In the MN assay, serial twofold dilutions of heat-inactivated serum in DMEM (1:10 to 1:320) were mixed (1:1) with 100 TCID_50_ of WNV and incubated for 1 h at 37 °C, 5% CO_2_. The serum/virus (50 µL) mixture was plated on Vero E6 cell monolayers (10^4^ cells/well) in the well of a 96-well plate and incubated for 1 h at 37 °C, 5% CO_2_. Then, 50 µL of DMEM was added to each well and the plate was incubated for 4 days up to the appearance of a cytopathic effect in control cultures (cell monolayers exposed to WNV). Serum negative for WNV was used as a control. The antibody titer was defined as the reciprocal of the highest dilution of the test serum sample, which showed at least 50% neutralization. A PRN assay was performed by exposing (1:1) serial twofold dilutions of heat-inactivated serum in DMEM (1:10 to 1:320) to 100 PFU of WNV. After incubation for 1 h at 37 °C, 5% CO_2_ atmosphere, 300 µL of the serum/virus mixture was plated on each well of 6-well plates seeded with 2.5 × 10^5^ Vero E6 cells and incubated 1 h at 37 °C. Then, the overlay medium composed of 0.5% Sea Plaque Agarose (Lonza, Basel, Switzerland) in propagation medium was added to each well. After 4 days of incubation at 37 °C, the monolayers were fixed with methanol (Carlo Erba Chemicals, Milan, Italy) and stained with 0.1% crystal violet (Carlo Erba Chemicals, Milan, Italy) and the viral titers were determined by PFU counting. The percentage of PRN was calculated by dividing the average PFU of viral serum-treated samples by the average of the viral positive control. All experiments were repeated at least twice. All experimental procedures were conducted under biosafety level 3 containment.

According to the EU case definition, a positive case was a subject with a WNV-specific antibody response in serum as an IgG titer and confirmation by neutralization [17].

### 2.5. Statistical Analysis

Statistical analyses were performed using SPSS software (version 14.0, Chicago, IL, USA) with parametric or non-parametric methods in the case of a non-normal data distribution. A *p*-value of less than 0.05 was considered statistically significant. The prevalence of WNV infection was calculated as the ratio between samples confirmed positive by PRN and all the tested samples. The 95% confidence interval of the prevalence was calculated using a binomial distribution. A Chi-square or Fisher test was used to evaluate the association between WNV positivity and specific demographic or occupational factors. A stepwise logistic regression analysis was used to evaluate the association between all the risk factors evaluated through the questionnaire and the WNV positivity.

## 3. Results

Table 1 presents the demographic and occupational characteristics of the 250 outdoor workers enrolled in the study.

Figure 1 illustrates the WNV seroprevalence rates across the investigated area. Table 2 shows the univariate analysis results for associations between WNV seroprevalence and occupational or demographic factors. Among the 250 serum samples tested, eight (3.2%) were positive for WNV by PRN and/or MN assays. Livestock breeders exhibited the highest WNV seroprevalence at 6.5%, whereas agricultural and forestry workers showed lower rates at 1.4% and 2.7%, respectively. Notably, no seropositive cases were identified among horse breeders or veterinarians, with no cases detected in the 40–59 age group. Other factors, including gender, other outdoor activities, and the geographical area of workers’ residence, working in wetland areas, specific animal contact, detecting dead birds near worksites, and PPE use, did not show significant associations with WNV seropositivity.

Table 3 provides a detailed breakdown of WNV seroprevalence across occupations, including ELISA IgG positivity, borderline IgG, and PRA/MN positivity. Livestock breeders exhibited higher rates, with 8.7% ELISA IgG positivity and 6.5% PRA/MN positivity. Overall, across all occupational groups, ELISA IgG positivity was 4%, borderline IgG was 0.8%, and PRA/MN positivity was 3.2%. WNV seroprevalence was assessed using a two-step serological approach. Initially, ELISA was performed to detect WNV-specific IgG antibodies. Samples that tested positive or borderline in ELISA were subsequently analyzed using PRN and MN assays to confirm WNV specificity. Due to the higher specificity of PRN/MN, some ELISA-positive samples were not confirmed as WNV-positive, leading to differences in positivity rates between techniques.

Table 4 summarizes the results of the stepwise logistic regression analysis, highlighting age 40–59 years, repellent use, and PPE usage as significant protective factors that reduced the likelihood of WNV infection among outdoor workers.

Table 5 details the eight confirmed WNV infection cases, including demographic and occupational characteristics, travel history, and serological test results. The cases included seven males and one female, aged 26 to 74 years, with most individuals reporting contact with animals, primarily cattle.

Of the eight positive subjects for WNV IgG antibodies who were further tested for Dengue, Zika, Chikungunya, and Toscana virus IgG, only one tested positive for Dengue virus. This individual, an Indian national, reported no travel in the past year and had been residing in Italy for approximately 2 years. However, this participant was not excluded from the study, as our questionnaire included specific questions regarding vaccination history, and the individual did not report having received a Dengue vaccine.

## 4. Discussion

This study examines the seroprevalence of West Nile Virus (WNV) among outdoor workers in various occupations in the Apulia region (Southern Italy), identifying distinct occupational and demographic factors linked to WNV infection. Our findings reveal that cattle breeders exhibited a slightly higher seroprevalence rate compared to other outdoor workers, suggesting that specific job characteristics may elevate the occupational risk of WNV infection.

Since the first human case of WNV infection was reported in Italy in 2007, the country has experienced a steady increase in both the geographical spread and the number of cases across different populations, including blood donors [11]. A key study conducted in Tuscany from 2016 to 2019 reported seroprevalence rates between 0.5% and 0.9%, indicating a broader circulation of WNV than previously recognized [18]. This aligns with the findings of Mencattelli et al. [19], whose comprehensive analysis of WNV lineage 2 in Italy, based on national surveillance data, highlights the progressive spread of the virus since its emergence. These studies underscore a worrying trend in Italy’s WNV epidemiological landscape.

In our study, we found a seroprevalence of 3.2%, which was significantly higher than that observed in the general population of the same area, particularly among cattle breeders with a seroprevalence of 6.5%. This suggests an increased work-related risk of WNV infection in this specific outdoor population. In addition, a recent study [20] indicates a low seroprevalence of WNV (0.32%) in blood donors from the Apulia region, suggesting that WNV circulation in the general population remains relatively limited. This rate is notably lower than the seroprevalence reported in occupationally exposed workers, such as livestock keepers and outdoor laborers in our study. The discrepancy in seroprevalence between these populations may be attributed to differences in exposure risk, as individuals engaged in outdoor professions experience more frequent mosquito bites and prolonged time spent in WNV-endemic environments.

In addition to human seroprevalence, several studies have focused on animal populations, particularly horses, which serve as important sentinels for WNV spread. Research across various Italian regions has shown a notable increase in seroprevalence among equids, reflecting the virus’s presence in the environment and its potential risk to both animal and human health [19,21]. While equids, like other mammals, are dead-end hosts and do not contribute to WNV transmission, their seroprevalence can provide valuable insights into the extent of viral activity in a given area. The integration of ecological and epidemiological data is critical for assessing WNV risk, especially considering changing climatic conditions that may influence vector populations and disease transmission patterns [21].

The seroprevalence of WNV among various occupational groups has become a growing concern due to the increasing incidence of WNV infections and the potential work-related exposure risks [5]. The observed seroprevalence of WNV among outdoor workers can be understood within the broader context of climate change and its impact on vector-borne diseases. In Europe, WNV has become a recurrent public health issue, with outbreaks rising in intensity, frequency, and geographic spread. This trend is largely driven by climate change, which creates more favorable conditions for the virus [22].

In recent years, climate change has notably impacted the climatic patterns of the Apulia region, resulting in a noticeable increase in average temperatures and shifts in rainfall distribution [23]. This warming trend has not only transformed local agricultural practices [24] but has also created conditions that promote the proliferation of mosquitoes, particularly the Culex species, which are primary vectors of WNV in the region [23]. The higher seroprevalence observed among outdoor workers in our study may be linked to these regional climate changes, which increase their exposure to mosquito bites due to the nature of their outdoor activities.

Occupational risk analysis indicates that livestock breeders face a higher risk of WNV exposure compared to other outdoor occupational groups, likely due to the nature of their work, which involves frequent exposure to mosquito-prone environments [25]. Man-made rural environments, such as livestock settings, may create ideal conditions for mosquitoes to thrive and interact with birds, wildlife, livestock, and humans, thereby increasing the risk of WNV outbreaks [26]. WNV circulation has been documented in cattle and sheep, with cases of lethal WNV encephalitis reported in several mammalian species, including ruminants [27]. Unlike birds, mammals cannot replicate WNV to infect mosquitoes, but they may serve as amplifying hosts for mosquito species with opportunistic feeding habits. Some Culex species, which are the most competent WNV vectors, feed on both birds and mammals [25]. This makes them bridge vectors capable of transmitting the virus from avian reservoirs to susceptible mammals, including humans. Given these dynamics, cattle and livestock, in general, could potentially act as sentinel species for monitoring the spread of WNV [28]. Molecular studies are crucial to confirm these findings and support enhanced prophylactic measures, such as implementing sustained disinfestation procedures in herds. As demonstrated by Odigie et al. [5], contact with animals may represent an occupational risk factor for WNV transmission, particularly in settings where rural and urban environments intersect. Notably, WNV has been isolated from both ixodid and argasid ticks, although their vector competence remains poorly understood, and the knowledge of this potential transmission route is limited. Of particular interest, soft ticks (the argasid species) can maintain the virus in vivo for over 3 months and have been shown to transmit it to mice [29], suggesting they may serve as a potential WNV reservoir. This represents a lesser-studied and not fully understood transmission pathway, which may have implications in occupational contexts involving livestock.

Additionally, as noted by Bin et al. [30] and supported by a case report by Fonseca et al. [31], contact with WNV-infected birds is another significant occupational risk factor. This risk may be further compounded by the potential for aerosol transmission, a pathway previously demonstrated experimentally [32]. Direct exposure to infected animals, particularly through contact with potentially infectious bodily fluids or aerosols, poses possible risks to workers. While viremia levels in humans and horses are typically too low to sustain mosquito-borne transmission, the viral load in tissues from fatal cases may be sufficient to facilitate transmission through alternative routes. For example, Venter et al. [33] reported that invasive autopsy procedures might increase the risk of mucosal exposure and subsequent infection.

Cattle sheds and similar livestock enclosures may play a significant role in facilitating mosquito breeding and habitation, thereby increasing the exposure risk for livestock keepers. The warm and humid microclimate inside these structures, combined with the constant presence of animals, creates favorable conditions for mosquito survival and reproduction. Studies have shown that *Culex pipiens*, the primary vector of WNV in Europe, thrive in environments with high moisture levels and organic debris, both of which are commonly found in livestock facilities [34]. Additionally, water accumulation in drinking troughs, irrigation channels, and manure pits provides suitable breeding sites for mosquito larvae, further contributing to vector abundance and persistence [34]. Given that *Culex pipiens* exhibit nocturnal feeding behavior, individuals spending prolonged hours in cattle sheds during nighttime activities—such as monitoring parturition or animal health—may experience a higher risk of WNV transmission. This occupational exposure risk may help explain the higher WNV seroprevalence reported among livestock breeders compared to blood donors [20]. The increased nighttime presence in animal shelters, where mosquitoes are most active, could lead to more frequent mosquito bites and sustained exposure to the virus [34]. While our study did not explicitly assess whether livestock keepers regularly stay overnight in cattle sheds, previous reports indicate that such practices are common, particularly during critical periods such as calving or disease monitoring [35]. This prolonged nighttime exposure, coupled with the high mosquito density in livestock environments, may represent a significant but underrecognized risk factor for WNV transmission, warranting further investigation through targeted surveys and enhanced vector control strategies in agricultural settings.

Unexpectedly, unlike cattle breeders, military horse breeders have not shown any cases of WNV infection, although there was a case of infection in one of the bred horses. This finding can be attributed to several factors. Civilian livestock breeders typically work in less regulated environments, unlike military facilities that adhere to stringent hygiene and veterinary protocols, which often include mosquito control measures designed to reduce vector populations and mitigate WNV exposure risks [36]. Additionally, military personnel are more likely to follow regulated health and safety practices, including the use of PPE and repellents, also due to high-risk knowledge and perception connected to their socioeconomic background [37]. These combined factors could have created a higher-risk environment for WNV exposure among the livestock breeders investigated.

Interestingly, the absence of cases among veterinarians may be attributable to the use of PPE, or adherence to veterinary biosafety protocols. Their professional training may also contribute to greater awareness and adherence to mosquito bite prevention strategies, including the use of PPE and insect repellents. Future research should further investigate these protective factors and assess whether similar trends are observed in other regions [38].

The stepwise logistic regression analysis identifies three significant variables: age (40–59 years), use of repellents, and use of PPE. The use of repellents and PPE emerges as a critical protective measure, emphasizing the importance of consistent application in high-risk environments as a key preventive strategy against WNV, further underscoring the need for robust workplace safety protocols [37].

Our study also revealed an age-related trend, with individuals aged 40–59 years exhibiting lower infection rates. This finding may be due to behavioral factors, such as greater adherence to protective measures, or variations in occupational tasks among different age groups. Further research is needed to explore potential age-related immunological responses or exposure patterns.

The study’s cross-sectional design limits its ability to establish causal relationships between exposure variables and WNV seropositivity. The use of serological testing also presents challenges. While ELISA and MN/PRN assays are commonly employed in flavivirus research, they are prone to PRN cross-reaction with other flaviviruses, such as the Usutu virus (USUV), which can lead to false positives. This cross-reactivity may result in an overestimation of WNV seroprevalence, especially among participants previously exposed to other flaviviruses. However, according to the most recent national surveillance reports, no cases of USUV infection were reported in the Apulia region in humans, animals, or vectors during the study period.

The absence of evidence for USUV circulation in this region suggests that cross-reactivity with USUV antibodies is unlikely to have significantly impacted our results. Furthermore, the confirmation of positive cases through MN and/or PRN assays enhances the specificity for WNV detection. While we acknowledge that the complete exclusion of cross-reactivity is challenging, the available epidemiological data strongly support the reliability of our findings [39,40].

Despite these limitations, our study offers valuable insights. The findings highlight livestock breeders as a potentially valuable sentinel population for monitoring the spread of WNV in the region. Preventive and educational programs that emphasize the proper use of PPE and insect repellents could be crucial in reducing WNV transmission risks, particularly among high-risk occupational groups.

## 5. Conclusions

Future longitudinal studies should be conducted to track seroprevalence and immune response over time in outdoor workers. Moreover, investigation into additional risk factors for WNV seropositivity in occupational settings is essential. Identifying alternative occupational transmission routes of WNV is critical for developing effective protective measures for at-risk workers. Moreover, exploring the potential expansion of WNV hosts to include species beyond birds is crucial, especially given that infections have been identified in unexpected hosts, such as reptiles.

Understanding these factors could support the development of more refined, evidence-based strategies tailored to high-risk worker groups, which would inform policy recommendations for outdoor occupational health in WNV-endemic regions.

## Figures and Tables

**Table 1:** Demographic and occupational characteristics of the study population.

Variables	Workers (N. 250)		
	N (%)	Median	Range
Age (years)		50.0	21–76
20–39	61 (24.4)		
40–59	137 (54.8)		
60–79	52 (20.8)		
Working seniority (years)		20.0	1–60
1–15	87 (34.8)		
16–30	94 (37.6)		
>30	69 (27.6)		
Sex (male/female)	209 (83.6)/41 (16.4)		
Occupation			
Agricultural workers	69 (27.6)		
Livestock breeders	92 (36.8)		
Horse breeders	31 (12.4)		
Forestry workers	37 (14.8)		
Veterinarians	21 (8.4)		
Outdoor working activity (hours/day)		8.0	3–12
Working in wetlands	80 (32.0)		
Animal working contact			
Cattle	127 (50.8)		
Sheep	105 (42.0)		
Horses	89 (35.6)		
Pigs	33 (13.2)		
Wild animals	4 (1.6)		
Domestic animal contacts			
Dogs	25 (10.0)		
Cats	24 (9.6)		
Livestock birth assistance	114 (45.6)		
Use of PPE/repellents at work	213 (85.2)/124 (49.6)		
Referred occupational contact with WNV-positive horse	10 (4.0)		
Detection of dead birds near the work site	46 (18.4)		
Living area			
Rural/Urban	115 (46.0)/135 (54.0)		
Presence of mosquito nets in the dwelling	201 (80.4)		
Hobbies			
Gardening	107 (42.8)		
Camping	10 (4.0)		
Hiking	24 (9.6)		
Hunting	12 (4.8)		
Travelling in the last year			
Italy	115 (46.0)		
Europe	56 (22.4)		
Out of Europe	15 (6.0)		
Vaccinations for YF, Dengue virus or TBE	5 (2.0)		

**Table 2:** Univariate analysis of the association of workers’ prevalence of WNV infection with different occupational and demographic factors.

Variable	Confirmed Positives/N (%)	*p*-Value	OR	C.I. 95%
Occupation				
Agricultural workers	1/69 (1.4)	0.45	0.36	0.04–3.02
Livestock breeders	6/92 (6.5)	0.05	5.44	1.07–27.6
Horse breeders	0/31 (0.0)	0.60	-	-
Forestry workers	1/37 (2.7)	1.00	0.81	0.09–6.84
Veterinarians	0/21 (0.0)	1.00	-	-
Sex				
Female	1/40 (2.5)	1.00	0.72	0.08–6.02
Age				
20–39	4/61 (6.6)	0.10	3.24	0.78–13.3
40–59	0/137 (0.0)	0.01	-	-
60–79	4/52 (7.7)	0.06	4.04	0.97–16.7
Working contacts with				
Sheep	4/105 (3.8)	0.45	1.39	0.34–5.71
Cattle	5/127 (3.9)	0.72	1.63	0.38–7.01
Horses	2/89 (2.2)	0.41	0.59	0.11–3.00
Pigs	2/33 (6.1)	0.28	2.26	0.43–11.7
Use repellents at work				
Yes	1/124 (0.8)	0.03	0.13	0.01–1.10
Use PPE				
Yes	5/213 (2.3)	0.09	0.27	0.06–1.19

**Table 3:** Seroprevalence for WNV in the study population divided by occupation.

Occupation	N. ELISA Positive IgG/N (%)	N. ELISA Borderline IgG/N (%)	N. PRN and/or MN Positive/N (%)
Agricultural workers	2/69 (2.9)	1/69 (1.4)	1/69 (1.4)
Livestock breeders	8/92 (8.7)	0/92 (0.0)	6/92 (6.5)
Horse breeders	0/31 (0.0)	0/31 (0.0)	0/31 (0.0)
Forestry workers	0/37 (0.0)	1/37 (2.7)	1/37 (2.7)
Veterinarians	0/21 (0.0)	0/21 (0.0)	0/21 (0.0)
Total	10/250 (4.0)	2/250 (0.8)	8/250 (3.2)

**Table 4:** Multiple stepwise logistic regression analysis of potential risk factors associated with WNV seropositivity in the study population.

Variable	β	B	C.I. 95%	SE	*p*-Value	OR
Age 40–59 years	−0.205	−0.072	−0.03 −0.10	0.022	<0.001	0.93
Use repellents at work	−0.145	−0.051	−0.01 −0.09	0.022	0.019	0.95
Use PPE	−0.124	−0.061	−0.001 −0.121	0.030	0.044	0.94
Model R2: 0.075 *p* < 0.01						

**Table 5:** Demographic and occupational characteristics in the WNV confirmed cases observed in the study population.

No	Gender	Age (Years)	Occupation	Animal Contact	Traveling Abroad	ELISA IgG	MN Titer	PRA Titer
1	F	60	Forestry worker	None	Slovenia, Austria, Lombardy, Emilia-Romagna, Tuscany	Borderline	<10	40
2	M	27	Livestock breeder	Cattle, horses, pigs	Lazio	Positive	40	80
3	M	31	Livestock breeder	Cattle, sheep	Germany	Borderline	<10	20
4	M	30	Livestock breeder	Cattle, sheep	Not reported	Positive	20	80
5	M	74	Livestock breeder	Cattle, sheep, horses, pigs	None	Positive	10	20
6	M	63	Livestock breeder	Sheep	None	Positive	40	80
7	M	74	Agricultural worker	None	Lazio	Positive	20	40
8	M	26	Livestock breeder	Cattle	None	Positive	20	20

## Data Availability

The datasets used and analyzed during the current study are available from the corresponding author on reasonable request.

## Abbreviations

The following abbreviations are used in this manuscript:

WNV	West Nile virus
ELISA	Enzyme-linked immunosorbent assay
PPE	Personal protective equipment
WNF	West Nile fever
WNND	West Nile neuroinvasive disease
TBEV	Tick-borne encephalitis virus
YFV	Yellow fever virus
JEV	Japanese encephalitis virus
MN	Microneutralization
PRN	Plaque reduction neutralization
DMEM	Dulbecco’s Modified Eagles Medium
FBS	Fetal Bovine Serum
TCID_50_	50% tissue culture infectious dose
PFU	Plaque-forming unit
